# Clinical application of sugammadex in renal impairment: pharmacokinetics and pharmacodynamics

**DOI:** 10.3389/fphar.2026.1932040

**Published:** 2026-09-02

**Authors:** Hao-Wei Lee, Pao-Shan Chen, Min-Jia Li, Chih-Shung Wong

**Affiliations:** Department of Anesthesiology, Cathay General Hospital, Taipei, Taiwan

**Keywords:** end-stage renal disease, pharmacodynamic, pharmacokinetic, renal impairment, sugammadex

## Abstract

Sugammadex, a modified γ-cyclodextrin, rapidly and predictably reverses aminosteroid neuromuscular blockade by directly encapsulating rocuronium and vecuronium, thereby avoiding the cholinergic adverse effects associated with acetylcholinesterase inhibitors. Because both free sugammadex and the sugammadex–rocuronium complex are eliminated predominantly through renal excretion, the use of sugammadex in patients with severe renal impairment remains a topic of debate. This review summarized current evidence on sugammadex by exploring its molecular mechanisms, pharmacokinetic and pharmacodynamic properties, clinical efficacy, and safety profile in patients with renal impairment. The literature indicates that severe renal impairment markedly prolongs the systemic retention of sugammadex and sugammadex–rocuronium. However, this pharmacokinetic alteration does not compromise the rapid reversal of neuromuscular blockade or increase the risk of recurarization or major adverse events. High-flux hemodialysis increases the clearance of sugammadex–rocuronium, but evidence supporting continuous renal replacement therapy and peritoneal dialysis remains limited. Overall, sugammadex may serve as an effective and generally well-tolerated reversal agent in carefully selected patients with severe renal impairment. Additional prospective studies are required to establish its long-term safety and guide clinical recommendations.

## Introduction

Neuromuscular blockade reversal is a key component of modern anesthetic practice. Traditional reversal agents, such as acetylcholinesterase inhibitors, exhibit variable efficacy and may lead to adverse events (e.g., bradycardia, hypersalivation, bronchoconstriction, and increased airway secretions) (Khuenl-Brady et al., 2010). To counteract these muscarinic adverse effects, neostigmine is routinely co-administered with anticholinergic agents such as atropine or glycopyrrolate. Sugammadex, a selective relaxant binding agent, offers a novel mechanism for the direct and rapid reversal of aminosteroid neuromuscular blockade (Welliver et al., 2008). It is a modified γ-cyclodextrin that forms a highly stable inclusion complex with aminosteroid neuromuscular blocking agents (Nag et al., 2013) and is eliminated almost exclusively through renal excretion. Several narrative reviews have summarized findings regarding the use of sugammadex in patients with renal impairment, particularly those with end-stage renal disease (ESRD). Recent pharmacokinetic studies and systematic reviews and emerging evidence on various dialysis modalities (e.g., high-flux hemodialysis, continuous renal replacement therapy, and peritoneal dialysis) have substantially expanded the literature. The present review provides an updated overview by integrating latest pharmacokinetic and pharmacodynamic evidence, data on dialysis-mediated drug elimination, and practical perioperative considerations to support clinical decision-making in renal impairment.

## Molecular structure and mechanism of action

**FIGURE 1:**
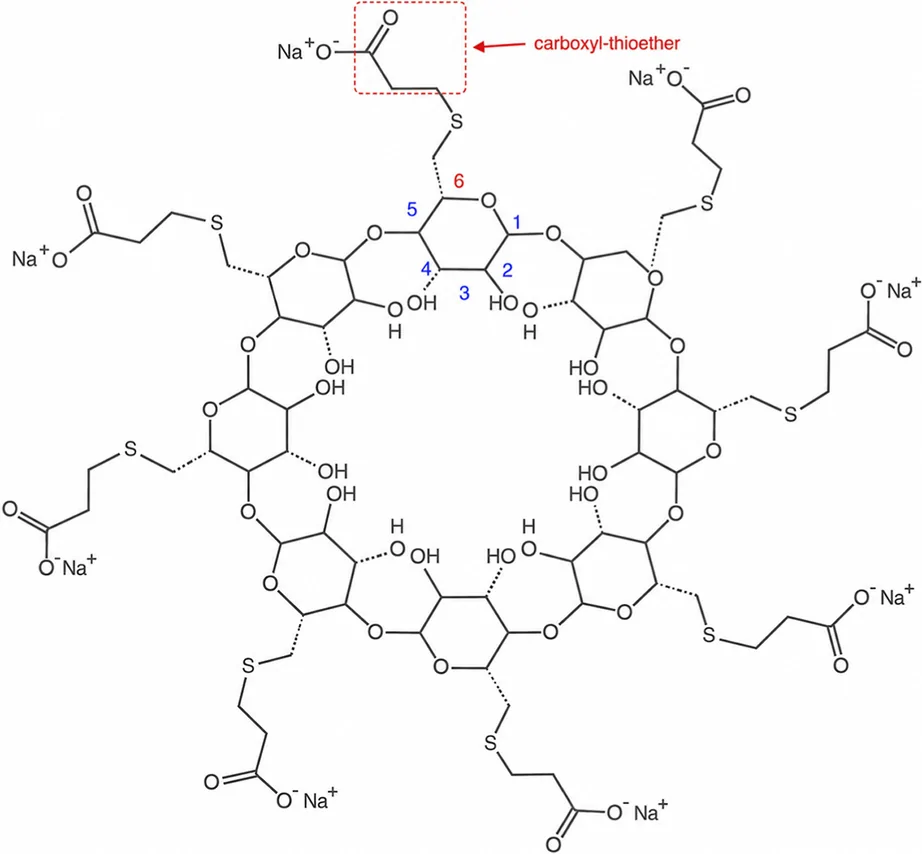
Structure of sugammadex. Adapted and modified from Mogane (2020).

Cyclodextrins are cyclic glucose polymers that consist of six (α-cyclodextrins), seven (β-cyclodextrins), or eight (γ-cyclodextrins) glucose units. These polymers have a truncated cone structure with a hydrophobic inner cavity and hydrophilic outer surface, which enable the encapsulation of lipophilic molecules. Sugammadex is a modified γ-cyclodextrin composed of eight glucose units, with carboxyl thioether side chains attached at the C6 position (Figure 1). These modifications enlarge sugammadex’s internal cavity and enhance its electrostatic interactions, enabling its high-affinity binding to aminosteroid neuromuscular blocking agents, particularly rocuronium (Srivastava and Hunter, 2009). Sugammadex binds to blocking agents at a 1:1 ratio and is stabilized through van der Waals forces, hydrogen bonds, and hydrophobic interactions (Nag et al., 2013; Adam et al., 2002). Its binding affinity varies across drugs, being the highest for rocuronium (∼95%), followed by vecuronium (∼90%) and pancuronium (∼61%). Sugammadex directly encapsulates free neuromuscular blocking agents in plasma, rendering them inactive. The reduction in the plasma concentrations of free blocking agents establishes a concentration gradient that drives the dissociation of these molecules from nicotinic receptors at the neuromuscular junction, leading to rapid recovery of muscle power (Adam et al., 2002; Mitchell and Lobaz, 2016).

Molecular weight is a key determinant of dialytic clearance. Vanholder et al. (2003) classified uremic retention solutes into small water-soluble molecules, protein-bound molecules, and middle molecules. They noted that middle molecules exhibit distinct clearance kinetics when used in extracorporeal therapies. Sugammadex is classified as a middle molecule (molecular weight: ∼2 kDa), whereas rocuronium is classified as a small water-soluble molecule (molecular weight: ∼610 Da). Together with protein-binding properties, these physicochemical characteristics explain the varying dialyzability of free sugammadex, free rocuronium, and the sugammadex–rocuronium complex. They also provide a mechanistic rationale for the preferential use of high-flux hemodialysis to promote sugammadex clearance in patients with ESRD (Vanholder et al., 2003).

## Pharmacokinetics and clearance

In healthy adults, sugammadex demonstrates linear pharmacokinetics over the recommended clinical dose range. Approximately 90%–95% of the administered dose is excreted unchanged in the urine within 24 h, with an elimination half-life of approximately 2 h. Because sugammadex undergoes minimal metabolism and negligible protein binding, renal function is the primary determinant of its systemic clearance. The highly stable sugammadex–rocuronium complex formed after encapsulation is also eliminated predominantly through renal excretion.

Rocuronium itself is primarily eliminated through biliary excretion, whereas both free sugammadex and sugammadex–rocuronium are eliminated almost exclusively by the kidneys (Staals et al., 2010). Only an extremely small fraction (∼1 in 25 million) of the sugammadex–rocuronium complex dissociates into free rocuronium, reflecting the high thermodynamic stability of the complex (Nag et al., 2013).

Although renal impairment substantially alters the pharmacokinetics of both sugammadex and rocuronium, it does not compromise the rapid reversal of rocuronium-induced neuromuscular blockade. No recurarization is observed at 48 h after the administration of sugammadex. However, the evidence is derived from small cohorts (Staals et al., 2010). Consistent with the classification proposed by Vanholder et al. (2003), molecules larger than 500 Da are classified as middle molecules, which exhibit clearance characteristics distinct from those of small water-soluble molecules. Because sugammadex is considered a middle molecule, it is eliminated predominantly through renal filtration in individuals with normal kidney function (Vanholder et al., 2003).

### Clinical dosing strategies

Sugammadex dosing depends on the depth of neuromuscular blockade, as determined through neuromuscular monitoring, and varies by the clinical scenario. Table 1 presents the recommended doses of sugammadex required to achieve a train-of-four (TOF) ratio of ≥0.9, which is considered the endpoint for adequate reversal.

**TABLE 1:** Sugammadex dosing strategy.

Clinical scenario	Neuromuscular monitoring	Dose	Time to TOF ≥0.9
Routine reversal	TOF count ≥2	2 mg/kg	∼2 min
Moderate block	PTC 1–2	4 mg/kg	∼3 min
Profound block	3–5 min post- neuromuscular blocking agents	16 mg/kg	∼1.5 min

TOF, Train-of-four; PTC, Post-Tetanic Count.

In a randomized controlled trial comparing sugammadex with neostigmine plus glycopyrrolate for the reversal of profound rocuronium-induced neuromuscular blockade (one to two post-tetanic counts), sugammadex (4 mg/kg) achieved recovery to a TOF ratio of 0.9 within a mean duration of 2.9 min, whereas neostigmine (70 μg/kg) plus glycopyrrolate required 50.4 min to achieve recovery (Jones et al., 2008). Therefore, sugammadex enabled approximately 17-fold faster reversal than did neostigmine plus glycopyrrolate and reliably achieved rapid recovery from profound neuromuscular blockade.

## Readministration of neuromuscular blocking agents

After the administration of sugammadex, higher doses of rocuronium may be required to re-establish neuromuscular blockade because residual circulating sugammadex can bind subsequently administered aminosteroid neuromuscular blocking agents. If neuromuscular blockade is required within 5 min after sugammadex administration, a higher dose of rocuronium (1.2 mg/kg) is recommended. After an interval of 4 h, 0.6 mg/kg rocuronium may be used. When more than 24 h have elapsed, standard rocuronium dosing can generally be administered without major concerns regarding interaction with residual sugammadex. Alternatively, nonaminosteroid agents, such as succinylcholine or benzylisoquinolinium, can be used to avoid this interaction (Chandrasekhar and Jeffers, 2019).

## Adverse effects

Although sugammadex is generally well tolerated, several adverse effects have been reported. Hypersensitivity reactions are among the most serious concerns because they can occur rapidly and become severe, typically within approximately 5 min after administration. Although the incidence rate of hypersensitivity reactions is less than 1%, the risk appears to increase with increasing doses and repeated exposure (Jahr et al., 2015). Community cyclodextrin exposure has been proposed as a mechanism of sensitization in cases where hypersensitivity occurs upon the first exposure (Crimmins et al., 2024). However, no robust data are currently available to support this hypothesis.

Sugammadex may affect coagulation by transiently inhibiting the activity of factor Xa, thereby temporarily prolonging activated partial thromboplastin time (Kam et al., 2014). It can also prolong the QTc interval, thereby predisposing susceptible patients to cardiac arrhythmias.

## Drug–drug interactions

Potential drug–drug interactions should be considered during the administration of sugammadex. These interactions can be broadly categorized into displacement reactions and capture reactions. Displacement reactions may theoretically occur when competing drugs, such as toremifene, fusidic acid, flucloxacillin, or diclofenac, prevent the binding of sugammadex to rocuronium. However, no clinically meaningful recurarization has yet been reported, likely because of the extremely high affinity and low dissociation rate of the sugammadex–rocuronium complex (Mitchell and Lobaz, 2016). Capture reactions occur when unbound sugammadex encapsulates other molecules with a structural affinity for its binding cavity, which reduces the pharmacological activity. The most clinically relevant example is oral contraceptives, whose efficacy may decrease following exposure to sugammadex. Therefore, additional contraceptive precautions are recommended for 7 days after sugammadex administration. Other drugs, such as cortisone and remifentanil, have also been reported to undergo capture reactions. However, current evidence suggests that these interactions have minimal clinical importance (Mitchell and Lobaz, 2016).

## Sugammadex versus neostigmine

Sugammadex achieves substantially faster recovery (up to 17 times faster) from neuromuscular blockade than does neostigmine (Jones et al., 2008). Compared with neostigmine plus an anticholinergic agent, sugammadex offers high hemodynamic stability by avoiding anticholinergic-related tachycardia and hypertension, exerting minimal effects on heart rate and blood pressure after reversal. Furthermore, sugammadex carries a low risk of postoperative nausea and vomiting, although studies have reported inconsistent results. By contrast, neostigmine, particularly at higher doses, has been consistently associated with a high risk of postoperative nausea and vomiting and increased postoperative use of antiemetic agents (Chang et al., 2022).

## Sugammadex use in special populations

Sugammadex is both safe and effective in patients with myasthenia gravis. It achieves reliable neuromuscular blockade reversal without increasing the risk of postoperative complications such as myasthenic crises, respiratory failure, reintubation, atelectasis, or pleural effusion (No et al., 2023). Sugammadex is also safe in patients with severe pulmonary diseases, such as bronchiectasis and cystic fibrosis. In these high-risk populations, rapid and reliable reversal of neuromuscular blockade may mitigate the risk of postoperative respiratory complications such as residual neuromuscular blockade, delayed extubation, reintubation, atelectasis, and pneumonia (Mitchell and Lobaz, 2016). However, patients with heart failure and those with a history of cardiac transplantation may experience prolonged recovery, likely because of reduced cardiac output and delayed drug distribution. A retrospective study revealed similar rates of bradycardia and hypotension between patients who received sugammadex and those who received neostigmine, suggesting that both agents can be used safely in this population (Paredes et al., 2023). Because mild to moderate hepatic impairment has minimal effects on the pharmacokinetics and clinical efficacy of sugammadex, dose adjustment is generally not required in patients with this condition.

## Sugammadex in renal impairment

Patients with renal impairment exhibit delayed plasma clearance of sugammadex. Therefore, systemic exposure area under the curve and elimination half-life are significantly higher but total plasma clearance is markedly lower in patients with severe renal dysfunction than in individuals with normal renal function. Despite the pharmacokinetic changes, the pharmacodynamic effect of sugammadex remains largely preserved because the reversal mechanism depends primarily on the rapid encapsulation of circulating rocuronium rather than on the renal elimination of the complex. Therefore, the recovery of neuromuscular transmission remains rapid, although it is slightly slower in patients with renal impairment than in individuals with normal renal function (Staals et al., 2008; Panhuizen et al., 2015).

According to the current US Food and Drug Administration Prescribing Information and the European Medicines Agency Summary of Product Characteristics, sugammadex is not recommended for patients with severe renal impairment (creatinine clearance <30 mL/min) because of delayed elimination and insufficient long-term safety data. However, accumulating clinical evidence suggests that sugammadex effectively reverses rocuronium-induced neuromuscular blockade even in patients with ESRD. In a multicenter study (Staals et al., 2008), sugammadex administration at two or 4 mg/kg resulted in rapid and predictable recovery consistent with the depth of neuromuscular blockade, with recovery to a TOF ratio of ≥0.9 occurring within approximately 2–3 min, although the recovery remained modestly slower than that in individuals with normal renal function. Nonetheless, the recovery was still substantially faster and more reliable than that historically reported with neostigmine, supporting the preservation of sugammadex’s pharmacodynamic efficacy despite impaired renal clearance.

Panhuizen et al. (2015) reported that sugammadex at 4 mg/kg successfully achieved deep neuromuscular blockade reversal in patients with severe renal impairment. Although the plasma concentrations of sugammadex and sugammadex–rocuronium remained high for a long period because of reduced renal clearance, recovery to a TOF ratio of ≥0.9 remained rapid, reproducible, and dose-dependent, with no evidence of clinically meaningful recurarization.

Historically, cisatracurium has often been preferred over rocuronium in patients with severe renal impairment because its organ-independent Hofmann elimination avoids the prolonged and unpredictable duration of action associated with rocuronium in this population. Accumulating clinical experience indicates that administering rocuronium followed by sugammadex represents a feasible alternative when rapid and reliable reversal is desired.

As mentioned, the rocuronium–sugammadex complex is highly stable. This stability is maintained by a combination of intermolecular (van der Waals) forces, thermodynamic (hydrogen) bonds, and hydrophobic interactions. Only approximately 1 in every 25 million complexes dissociates to release free rocuronium, which indicates the extremely high stability of the rocuronium–sugammadex complex. The complex formed after encapsulation is highly water-soluble and eliminated predominantly through renal excretion. Severe ESRD substantially reduces clearance of the sugammadex–rocuronium complex, thereby prolonging its retention in the circulation. However, the long-term disposition of the complex and its clinical implications remain unclear. Despite the delayed elimination, no clinical case of recurarization has been reported, reflecting the exceptionally high binding affinity of sugammadex for rocuronium, which renders the dissociation of the complex highly unlikely (Staals et al., 2010). The sugammadex–rocuronium complex can be effectively removed through high-flux hemodialysis, which offers an effective approach for encapsulated complex clearance in patients with severe renal impairment. The dialyzability of the complex may partly explain the low incidence of recurarization observed in patients with advanced renal dysfunction despite delayed clearance of the sugammadex–rocuronium complex (Oh and Kim et al., 2023).

## Systematic review and meta-analysis involving patients with ESRD

Kim et al. (2021) conducted a systematic review and meta-analysis of studies evaluating the efficacy and safety of sugammadex in patients with ESRD. They divided the included studies into two groups: Group R (estimated glomerular filtration rate <30 mL/min/1.73 m^2^) and Group N (estimated glomerular filtration rate ≥80 mL/min/1.73 m^2^). The outcomes (drug clearance, neuromuscular recovery, and adverse events) were analyzed from both pharmacokinetic and clinical perspectives. Kim et al. reported that ESRD significantly altered the pharmacokinetic profiles of rocuronium and sugammadex, reducing their clearance. Recovery from neuromuscular blockade, evaluated in terms of the time required to achieve a TOF ratio of 0.9, was slower in Group R than in Group N. However, no significant between-group difference was observed in the incidence of prolonged recovery to a TOF ratio of 0.9. Furthermore, no significant difference was observed in the incidence of recurrent neuromuscular blockade between patients with ESRD and individuals with normal renal function. Pharmacokinetic analysis in Group R revealed that clearance was higher for rocuronium than for sugammadex. Thus, the circulating concentration of sugammadex exceeded that of rocuronium, maintaining the encapsulation of free rocuronium molecules and minimizing the risk of recurarization. This pharmacokinetic relationship may explain why the rate of neuromuscular blockade recurrence did not increase despite the impairment of renal function. Kim et al. (2021) further noted markedly increased plasma concentrations of rocuronium at 12 h after administration in patients with ESRD and attributed this finding to the measurement of both free rocuronium and encapsulated rocuronium within the rocuronium–sugammadex complex. This interpretation may explain why high plasma concentrations of rocuronium were observed without a corresponding increase in the incidence of recurrent neuromuscular blockade.

Despite the aforementioned findings, major uncertainties remain. For example, clinical data on the long-term disposition, tissue distribution, and ultimate fate of the rocuronium–sugammadex complex in patients with ESRD remain lacking. Therefore, although current evidence suggests that sugammadex can effectively reverse neuromuscular blockade without increasing the risk of recurarization in patients with advanced renal dysfunction, whether the rocuronium–sugammadex complex can be safely and fully eliminated in patients with ESRD remains unknown.

In patients undergoing high-flux hemodialysis, approximately 69% of circulating sugammadex and 75% of rocuronium can be removed during the first dialysis session. Subsequent sessions can eliminate approximately 50% of the remaining drug burden. Therefore, in patients undergoing chronic dialysis, hemodialysis should be performed within 24–48 h after surgery to facilitate clearance of the rocuronium–sugammadex complex. However, evidence supporting the efficacy of low-flux hemodialysis in eliminating free sugammadex or rocuronium–sugammadex remains limited.

Overall, current evidence suggests a favorable safety profile for sugammadex in patients with ESRD. Regarding postanesthetic adverse outcomes, no increase has been reported in the incidence of recurrent neuromuscular blockade or prolonged recovery to a TOF ratio of 0.9. Likewise, no major laboratory abnormalities have been reported, and no clinically meaningful hemodynamic instability has been observed. These findings support the short-term efficacy and safety of sugammadex in patients with advanced renal dysfunction. Nonetheless, additional studies are required to clarify its long-term disposition and safety profile. Collectively, the findings suggest that delayed pharmacokinetic elimination does not necessarily translate into impaired pharmacodynamic efficacy, which highlights the dissociation between drug disposition and clinical reversal in patients with renal dysfunction.

## Conclusion

Sugammadex is an effective and generally well-tolerated reversal agent for neuromuscular blockade in patients with severe renal impairment, including carefully selected patients with ESRD. Despite the markedly prolonged systemic retention of the sugammadex–rocuronium complex, current clinical evidence suggests no increase in the risk of recurarization or any other major adverse outcomes. High-flux hemodialysis effectively removes the sugammadex–rocuronium complex, but evidence supporting continuous renal replacement therapy and peritoneal dialysis remains limited. The long-term disposition of this complex and its clinical implications remain unclear. Additional large-scale prospective studies with longer follow-ups are required to better define the long-term safety profile of the sugammadex–rocuronium complex and guide clinical recommendations for patients with advanced renal dysfunction.
